# Effect of External Teat Sealant on the Prevention of Intramammary Infection for Milking Cows: A Randomized Cross-Over Design Study

**DOI:** 10.3390/microorganisms13040819

**Published:** 2025-04-03

**Authors:** Yasunori Shinozuka, Takuya Kanda, Keiichi Hisaeda, Akira Goto, Yoichi Inoue, Naoki Yamamoto

**Affiliations:** Faculty of Veterinary Medicine, Okayama University of Science, Imabari 794-8555, Ehime, Japan; ta-kanda@ous.ac.jp (T.K.); k-hisaeda@ous.ac.jp (K.H.); a-goto@ous.ac.jp (A.G.); y-inoue@ous.ac.jp (Y.I.); n-yamamoto@ous.ac.jp (N.Y.)

**Keywords:** external teat sealant, intramammary infection, milking cow, teat end

## Abstract

This study clarified the effectiveness of external teat sealant (ETS) in preventing intramammary infections during lactation, using a cross-over study of two experiments (3 cows × 2 periods each) on a dairy farm. In Experiment 1, the control (Group A) received pre-dip and post-dip treatments, while the experimental group (Group B) received ETS application instead of post-dip treatment. In Experiment 2, Group C was treated the same as Group B, and Group D received ETS treatment only. After the intervention, teat ends were tested using ATP swabs, and milk collections from the first and last foremilk (Samples 1 and 2, respectively) were conducted over 4 days (8 times in total). In Experiment 1, the ETS application group exhibited lower ATP (*p* < 0.01) and bacterial counts (BC1, *p* = 0.02) compared to the control. Conversely, no differences in variables were observed in Experiment 2. The isolation rate of *Staphylococcus* spp. (>500 colony forming units) from Sample 2 in Groups C and D was significantly higher than that in groups A and B (*p* < 0.01). Replacing post-milking teat disinfection with ETS does not decrease viable bacterial counts and actually increases the proportion of *Staphylococcus* spp. ETS application is thus not an effective substitute for teat orifice disinfection.

## 1. Introduction

Bovine mastitis is the most common disease in dairy farms, affecting both animal welfare and the profitability of the dairy industry [1]. Most mastitis-causing microorganisms are bacteria that enter the mammary gland through the teat canal [2]. Consequently, mastitis is the primary reason for antibacterial agent use in dairy cows, with systemic and/or intramammary administration being the main treatment approach [3]. The widespread use of antibacterial agents to treat mastitis is also a serious public health concern, mainly due to increases in drug resistance [4] and the risk of residual agents in milk [5]. Consequently, the prevention of mastitis is an extremely important issue.

An effective method for preventing mastitis is post-dipping, in which the teats are immersed in a disinfectant immediately after milking to eliminate mastitis-causing bacteria on the teat skin [6]. Although post-dipping is considered an important measure for preventing contagious mastitis by removing bacteria introduced during milking [7], the disinfection effect is temporary (1–2 h) [8]. Consequently, it is ineffective against environmental microorganisms that adhere to the teat end between milkings [9]. In recent years, the incidence of contagious mastitis, such as that caused by *Staphylococcus aureus*, which originates from infected quarters, has decreased, while environmental mastitis, such as that caused by *Streptococci* and *Escherichia coli*, which originate from the environment, has been increasing [1]. Environmental mastitis develops when the causative bacteria penetrate and invade the teat canal from the contaminated teat canal between milkings, often due to the mechanical impact on the teat end during the milking process [9]. A correlation between udder hygiene scores and the risk of intermammary infection has been reported [10], highlighting the importance of preventing teat contamination before milking to reduce mastitis risk. One method for assessing contamination by organic matter or bacteria is the total adenylate (adenosine triphosphate (ATP) + adenosine diphosphate (ADP) + adenosine monophosphate (AMP)) hygiene monitoring test. In this assay, organic matter collected with a cotton swab reacts with a reagent to produce bioluminescence, and the emitted light is measured and quantified [11]. ATP bioluminescence assays using swabs from the teat orifice are also useful to assess pre-milking sanitation in lactating dairy cows [12]. Another key measure is the elimination of the causative bacteria at the teat orifice immediately before milking. A disinfection method known as pre-dipping has been shown to reduce the incidence of clinical mastitis by 50% [13]. Although pre-dipping is effective in preventing mastitis, its application immediately before milking raises public health concerns regarding potential disinfectant residues in the milk if wiping after disinfection is inadequate [5].

Internal and external teat sealants (ITS and ETS, respectively) function by physically blocking the teat canal, thereby preventing bacterial entry. The effectiveness of ITS, which are used to prevent new infections during the dry period [14], has the combined efficacy of ETS with antibacterial agents [15]. In addition, the barrier characteristics of various ETSs to prevent bacterial penetration under in vitro conditions have been reported [16]. However, to the best of our knowledge, the effectiveness of applying ETS to prevent intramammary infection in dairy cows has not yet been experimentally examined.

We hypothesized that ETS application after each milking would be more effective in preventing intramammary infection in Holstein cows than conventional teat disinfection methods such as pre- and post-dipping. Accordingly, this study aimed to clarify two hypotheses: (1) ETS can be used a substitute for post-dipping, and (2) ETS can be a substitute for both pre- and post-dipping. To assess these hypotheses, we conducted a cross-over study measuring teat orifice ATP as an indicator of pre-milking sanitation, bacterial counts in foremilk as a measure of bacterial infection, and somatic cell counts (SCC) immediately before milking.

## 2. Materials and Methods

The research protocol for this study was approved by the Okayama University of Science Experimental Animal Committee (Approval No.: Jitsu 2024-072). The farmer was informed about the purpose and the methods of the study, and oral consent was obtained from the farmer before commencement of the study.

### 2.1. Farm and Animals

This study was conducted on a dairy farm located in Kagoshima Prefecture in southern Japan in December 2024. The farm managed 700 Holstein cows housed in a free-stall barn and fed a total mixed ration (TMR) diet. The average milk yield per cow was 40 kg/day. The TMR diet comprised 15.3% crude protein (CP), 40.41% non-fibrous carbohydrates (NFC), and 34.53% crude fiber (CF). Milking was automated and performed three times daily at 5:00 AM, 12:00 PM, and 7:00 PM in an eight-cow double parallel parlor. Teat disinfection involved a pre-dip using a 0.15% effective iodine solution, SiLKY DiP^®^ (Umai Chemical Co., Tokushima, Japan), and a post-dip with a 1% effective iodine solution (Bovadine^®^ A; DeLaval International AB, Tumba, Sweden).

The quarters of the test cows were required to meet the following three conditions: (1) a milk SCC of <200,000 cells/mL, as determined using an automated somatic cell counter (ADAM^TM^ SCC; NanoEntek Co., Seoul, Republic of Korea) according to the manufacturer’s instructions; (2) a live bacteria count in milk of <1000 colony forming units (CFU)/mL, measured using a CompactDry^TM^ TC (Shimadzu Diagnostics Corp., Tokyo, Japan) according to the manufacturer’s instructions; and (3) the absence of *S. aureus*, confirmed by milk culture with CompactDry^TM^ X-SA (Shimadzu Diagnostics Corp.). A total of 24 healthy, mid-lactation, pregnant Holstein cows were screened, and based on the results, 12 cows (41 quarters) were selected for the study.

### 2.2. Study Design and Interventions

The 12 selected cows were assigned to Experiment 1 (6 cows, 19 quarters) and Experiment 2 (6 cows, 22 quarters). Both experiments followed a cross-over design with two periods. In Experiment 1, each cow received either post-dipping (Treatment A, control) or ETS application (Treatment B) immediately after milking in each period. Pre-dipping before milking was performed in both groups during both periods. In Experiment 2, each cow received either pre-dipping (Treatment C) or no disinfection before milking (Treatment D) throughout both periods. ETS application after milking with no post-dipping was conducted during both periods in both groups. Each period in both experiments consisted of six treatments over two days, with no wash-out period, as ETS has no residual effect. Samples were collected twice daily (7:00 AM and 7:00 PM). During sample collection, cows were first placed in the parlor, and ETS was removed (except in Treatment A). Teat-end swab samples were then collected immediately. Next, two milk samples were collected aseptically. The first sample (Sample 1) was obtained from the first foremilk before pre-dipping, except in Treatment D, where dipping was not performed. After sufficient foremilking to detect abnormalities and to initiate milk let-down, the second sample (Sample 2) was collected just before the milking unit was attached.

### 2.3. External Teat Sealant

**Figure 1:**
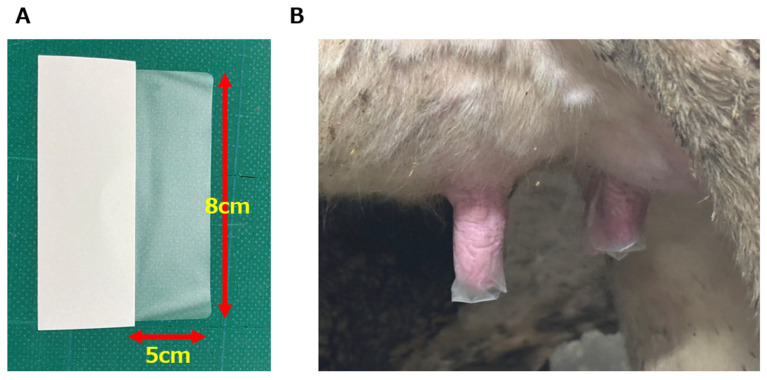
(**A**) The appearance of external teat sealant (ETS) before use. On the left is the backing (white), and on the right (translucent) is the part adhered to the teat when in use (5 × 8 cm^2^ ). (**B**) The appearance of the teat with the ETS adhered after milking. The ETS was left in place until the next milking, and removed by pinching its end with the fingers and pulling it down completely just before forestripping.

The ETS applied to the teat was 5 × 8 cm^2^ in size, and was made from plastic film and adhesive, using sanitary materials already on the market (Figure 1A). The ETS was applied by placing its center over the end of the teat after milking, then wrapping it with the hand to make it adhere tightly (Figure 1B). Just before the next milking, the ETS was removed by pulling its end downwards and peeling it off completely.

### 2.4. Teat Callosity

The degree of keratinization at the teat orifice was evaluated using the teat scoring method described by Mein et al. [17]. Briefly, before milking on the first day, the formation of a callosity ring and keratin protrusions at the teat orifice of each selected quarter were visually evaluated by a single veterinarian. The assessment was performed using the following four-level scale: 1 = no callosity ring present; 2 = callosity ring present but without roughness; 3 = callosity ring and roughness present, keratin fronds extending 1–3 mm from the teat orifice; and 4 = callosity ring present with excessive keratin fronds extending ≥4 mm from the teat orifice [17].

### 2.5. Teat Swabs

To assess teat-end contamination, the teat end was wiped with a moistened cotton swab using a LuciPack^®^ A3 Surface kit (Kikkoman Biochemifa Co., Tokyo, Japan) before milking. The swabbing technique was performed by two veterinarians using a standardized technique. The teat-end swabs were immediately analyzed for teat-end cleanliness using ATP luminometry with a Lumitester Smart^®^ kit (Kikkoman Biochemifa Co.) according to the manufacturer’s instructions.

### 2.6. Milk Tests

#### 2.6.1. Somatic Cell Counts

The SCC of Sample 2 was measured immediately after milk collection using the automated somatic cell counter ADAM^TM^ SCC described above.

#### 2.6.2. Bacterial Counts and Identification

The determination of CFUs was performed using the spread plate method. Briefly, 10 µL of each milk sample was plated onto AccuRate™ sheep blood agar (Shimadzu Diagnostics Corp.) within 12 h of sampling. The inoculated agar plates were then incubated at 37 °C for 24 h, after which the CFU per milliliter was determined. Five or more morphologically similar colonies on a plate were then isolated by transferring them to fresh blood agar and incubating for 24 h at 37 °C. Bacterial identification was then performed by matrix-assisted laser desorption ionization time-of-flight mass spectrometry (MALDI-TOF-MS) with the Biotyper system (Library BDAL11897_MycoV7 Bruker Daltonics, Bremen, Germany) [18]. This process was outsourced to the Ehime University Advanced Research Support Center (Ehime, Japan, https://www.adres.ehime-u.ac.jp/ accessed on 10 January 2025). In instances where the score was ≥2.0, the genus of the isolated bacteria was assigned to the top-ranked candidate. If the score was ≥1.5, then the genus was determined based on the lowest-ranked candidate appearing at least five times on the candidate list. Bacterial identification results were classified at the genus level, and taxa with an isolation rate of less than 3% were collectively classified as “Other”.

### 2.7. Statistical Analyses

All statistical analyses were performed using the R software package (v 4.4.2; https://www.r-project.org/ accessed on 3 February 2025). Model assumptions of normality were verified by logarithmic transformation of ATP and viable cell counts. The post-intervention results, including ATP levels, SCC, bacterial counts (BC) from Sample 1 (BC1) and BC from Sample 2 (BC2), were used as objective variables. First, to evaluate the carryover effect, an independent *t*-test was conducted between sequences. If a carryover effect was confirmed, either an independent *t*-test or analysis of variance (ANOVA) was performed. If no carryover effect was detected, data were analyzed using a mixed-effects model, with teat score, parity, sequence, period, and treatment as fixed effects, and individual quarters as random effects. The model was structured as follows:

Objective variable = β0 + β1·teat score + β2·parity + β3·sequence + β4·treatment + β5·period + β6·(treatment × period) + (1|quarter_id)

In Experiment 2, parity was not included as a fixed effect because all samples were from cows with a parity of 1. If a period effect was detected in the mixed-effects model analysis, an independent *t*-test was conducted using only the results from Period 1. The proportion of bacteria isolated at concentrations over 500 CFU/mL in each treatment from Sample 1 and Sample 2 was analyzed by ANOVA. A *p*-value of <0.05 was considered significant, except for period effects, where a threshold of 0.1 was used in the mixed model analysis.

## 3. Results

### 3.1. Overview of Enrolled Cows and Interventions

Table 1 presents the characteristics of the cows in Experiment 1 and 2, along with a summary of the interventions employed. In the cross-over study, the mean ± SD for parity and pre-intervention SCC of quarters were 1.1 ± 0.4 and 4.76 ± 4.60, respectively, in Experiment 1, and 1.0 ± 0.0 and 4.70 ± 4.46, respectively, in Experiment 2. The distribution of teat scores among the target quarters differed significantly between Experiment 1 and Experiment 2 (*p* < 0.01; Fisher’s exact test). The dropout rates (out of eight post-intervention sessions) for the ETS-applied treatment groups were 14.5% (11/76) in Treatment B, 30.7% (27/88) in Treatment C, and 23.9% (21/88) in Treatment D. Data from teat swabs and milk samples collected from quarters where the ETS had fallen off before milking were excluded from the analysis. As a result, the final analysis dataset included 76 quarters in Treatment A, 65 in Treatment B, 61 in Treatment C, and 67 in Treatment D. No cows exhibited systemic or local abnormalities or abnormal milk during clinical observations throughout the experimental period.

### 3.2. Outcomes

The results of each variable (ATP, SCC, BC1, and BC2) after Treatments A, B, C, and D are shown in Table 2.

The results obtained by mixed-effects model analysis using these results as the dependent variable are shown in Table 3.

In Experiment 1, the ATP of Treatment B was significantly lower (β = −0.43, SE = 0.12, t = −3.58, *p* < 0.01) than Treatment A. The random intercept and residual S.D. were 0.05 and 0.37, respectively. The AIC was 57.9. In SCC, the period had a significant effect (β = −0.11, SE = 0.05, t = −2.29, *p* = 0.04), and the random intercept and residual S.D. were 0.65 and 0.15, respectively. The AIC was 54.3. A paired *t*-test using data from Period 1 alone showed no difference (*p* = 0.34) between Treatment A and Treatment B. In BC1, Treatment B was significantly lower than Treatment A using a paired *t*-test based on Period 1 data because the carryover effect was confirmed by an independent *t*-test (*p* < 0.01) in advance. In BC2, the teat score (β = 0.67, SE = 0.27, t = 2.51, *p* = 0.02) and the period (β = −0.51, SE = 0.23, t = −2.18, *p* = 0.04) had a significant effect, respectively. The random intercept and residual S.D. were 0 and 0.72, respectively. The AIC was 99.5. A paired *t*-test using data from Period 1 alone showed no difference (*p* = 0.17) between Treatment A and Treatment B.

In Experiment 2, the period had a significant effect (β = 0.25, SE = 0.08, t = 3.27, *p* < 0.01) on ATP. The random intercept and residual S.D. were 0.17 and 0.26, respectively. The AIC was 43.4. A paired *t*-test using data from Period 1 alone showed no difference (*p* = 0.25) between Treatment C and Treatment D. In SCC, the carryover effect was confirmed by an independent *t*-test (*p* < 0.001) in advance. Although the SCC of teat score 3 was significantly lower than that of scores 1 and 2 using ANOVA, no significant difference (*p* = 0.11) was observed between Treatment C and Treatment D using a paired *t*-test based on Period 1 data. In BC1, the period had a significant effect (β = 0.54, SE = 0.23, t = 2.30, *p* = 0.03). The random intercept and residual S.D. were 0.40 and 0.78, respectively. The AIC was 125.8. A paired *t*-test using data from Period 1 alone showed no difference (*p* = 0.49) between Treatment C and Treatment D. In BC2, the period had a significant effect (β = 0.39, SE = 0.21, t = 1.89, *p* = 0.07). The random intercept and residual S.D. were 0.21 and 0.68, respectively. The AIC was 110.8. A paired *t*-test using data from Period 1 alone showed no difference (*p* = 0.78) between Treatment C and Treatment D.

#### 3.2.1. ATP

In Experiment 1, the fixed effect of ATP was significantly lower in Treatment B than in Treatment A (*p* < 0.01; Table 3). In Experiment 2, a significant period effect was observed (*p* < 0.01), and an alternative *t*-test showed no significant differences between treatment groups.

#### 3.2.2. Somatic Cell Counts

In Experiment 1, a significant period effect was observed (*p* = 0.04), but alternative *t*-tests did not reveal any significant differences between treatment groups. In Experiment 2, a carryover effect was observed before entering the mixed-effects model, so all variables were analyzed using Period 1 results only. The ANOVA test showed that SCC in quarters with a teat score of 3 was significantly lower than in those with teat scores of 2 and 1. However, independent *t*-tests showed no significant differences between treatment groups.

#### 3.2.3. Bacterial Counts in Sample 1 (BC1)

In Experiment 1, a carryover effect was observed, and an paired *t*-test showed that BC1 was significantly lower in Treatment B than in Treatment A (*p* = 0.02). In Experiment 2, a significant period effect was observed, but an alternative *t*-test did not show any significant differences between treatment groups.

#### 3.2.4. Bacterial Counts in Sample 2 (BC2)

A significant period effect in BC2 was observed in both Experiment 1 (*p* = 0.04) and Experiment 2 (*p* = 0.07). Consequently, an paired *t*-test was performed instead, but no significant differences were observed between treatment groups in either experiment. In Experiment 1, a fixed effect of teat score was observed (*p* = 0.02).

### 3.3. Bacterial Identification

**Figure 2:**
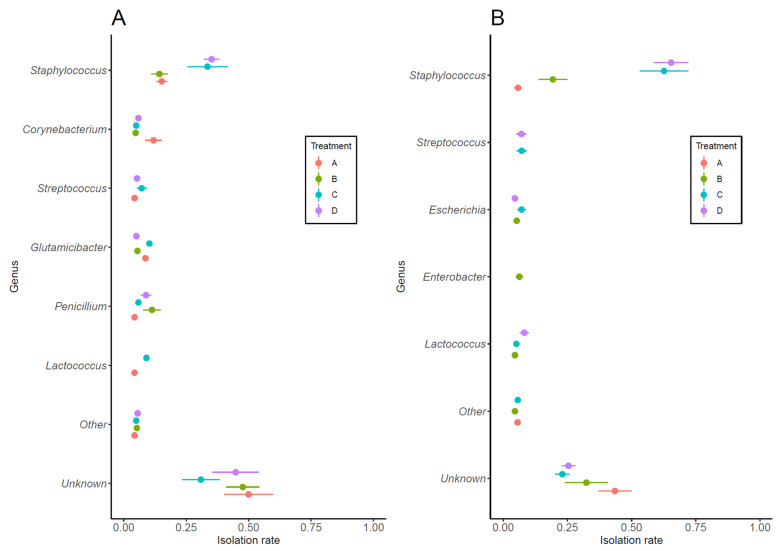
Bacteria isolated at ≥500 CFU/mL from Sample 1 (**A**) and Sample 2 (**B**) and their isolation rates. Bacteria were classified to genus, with those having an isolation rate of <3% collectively classified as “Other”. A significant difference in the isolation rate of *Staphylococcus* spp. in Sample 2 (**B**) was observed between Treatment A and Treatment C (AC), Treatment A and Treatment D (AD), Treatment B and Treatment C (BC), and Treatment B and Treatment D (BD) (*p* < 0.01 for each comparison; ANOVA with Bonferroni correction). Symbols and horizontal whiskers indicate means values and standard errors, respectively.

A total of 832 strains (393 from Sample 1 and 439 from Sample 2) were isolated from milk samples exhibiting bacterial growth of ≥500 CFU/mL. Of these, 169 from Sample 1 and 264 samples from Sample 2 were successfully identified, yielding a total of 433 identified samples, and 399 samples that remained unidentified. Figure 2 shows the bacteria that could be identified to genus level and their isolation rate in each treatment group. In Sample 1, the most frequently isolated bacteria were *Staphylococcus* spp., which showed a tendency to be more common in Treatment C and Treatment D, although no significant differences were observed between treatment groups. While the detection rate of *Corynebacterium* spp. tended to be higher in Treatment A, the difference was not statistically significant (Figure 2A). In Sample 2, *Staphylococcus* spp. were also the most frequently isolated bacteria. The isolation rate in Treatment A was significantly different from that in Treatment C (*p* < 0.01) and Treatment D (*p* < 0.01), with similar results obtained for Treatment B. The isolation rates of other bacterial genera were low, with no significant difference between treatment groups (Figure 2B).

## 4. Discussion

This study examined whether post-milking ETS application could serve as an alternative method of pre- and post-milking teat disinfection by preventing teat contamination.

In Experiment 1, we investigated whether ETS application could replace post-dipping. The results showed that ETS application significantly reduced ATP levels at the teat end just before milking. ATP quantification on various surfaces has been proposed as an indirect measure of bacterial contamination [19], and ATP monitoring is widely used as a standard hygiene assessment tool in the medical and food industries [18]. In addition, the ATP assay has also been used in the dairy industry to assess the effectiveness of teat cleaning procedures [20]. It is well established that environmental mastitis-causing bacteria can invade the udder at milking through a contaminated teat orifice between milking [9], and that maintaining teat cleanliness before milking is an effective strategy for preventing environmental mastitis [9]. In this study, the viable bacterial counts in Sample 1 (BC1) was significantly higher in Treatment A than in Treatment B. Additionally, in bacterial cultures of Sample 1, Treatment A, which did not employ ETS application, showed a tendency for a higher percentage of quarters from which *Corynebacterium* spp. was isolated, although this difference was not significantly different. Sample 1 was collected from the first foremilk of the previous milking and is considered to reflect the condition of the teat orifice. This suggests a possible relationship between teat contamination and the presence of *Corynebacterium* spp. On the other hand, no differences were observed between treatment groups in the proportion of quarters from which *Staphylococcus* spp., which was the most frequently isolated bacteria at ≥500 CFU/mL. This finding suggests that the source of *Staphylococcus* spp. may not be directly associated with teat contamination. Although BC2 did not differ significantly between treatment groups, the bacterial composition showed distinct trends. For example, the isolation frequency of *Staphylococcus* spp., a possible causative agent of environmental mastitis, tended to increase in the Treatment B group compared to Sample 1, whereas in Treatment A, it decreased. The viable bacterial counts in BC2 was approximately 1000 CFU/mL, which is within the normal milk threshold [21], and SCC counts remained below 300,000 cells/mL, which is the benchmark for intramammary infection [22]. However, the bacterial composition differed between Treatment A and Treatment B. These findings suggest that the absence of post-dipping may have altered the bacterial composition in a manner that could increase the risk of mastitis. While post-milking disinfection has long been recognized as an effective method for preventing new intramammary infections [6], the results indicate that ETS application cannot be considered as a substitute for post-dipping.

In Experiment 2, we tested whether ETS could fully replace teat disinfection before and after milking. The results showed no significant differences in ATP, SCC, BC1, or BC2 between Treatment C and Treatment D. Although BC2 was higher than in BC1 in both groups, SCC remained below 200,000 cells/mL in both groups, indicating the absence of intramammary infection [22]. However, BC2 exhibited an increased proportion of *Staphylococcus* spp., which includes potential mastitis-causing bacteria and suggests that omitting teat disinfection may increase the risk of intramammary infection. Interestingly, the isolation rate of *Staphylococcus* spp. differed significantly between Treatment B (Experiment 1) and Treatment C, despite both groups receiving the same intervention. Since the pre-milking procedure for both treatment groups included the removal of abnormal milk containing bacteria, the observed difference in *Staphylococcus* spp. isolation rates was considered to be attributable to experimental design. Specifically, while Treatment B received post-dipping as a pretreatment in both Period 1 and Period 2, the Treatment C group underwent post-dipping as a pretreatment only in Period 1 and not in Period 2. This difference may have influenced the isolation rate of *Staphylococcus* spp. between Group B and Group C. Additionally, the proportion of milk samples from Sample 2 containing ≥500 CFU/mL was significantly higher in Treatment C and Treatment D than in Treatment A and Treatment B (*p* < 0.01, respectively). In contrast, the bacterial isolation rate in the other groups decreased in Sample 2 compared to Sample 1. Sample 2 was collected after sufficient foremilking, just before the milking unit was attached, and thus reflects the quality of the milk intended for shipment. Although the SCC of this milk was <200,000 cells/mL, which is the benchmark for intramammary infection [22], the high isolation rate of *Staphylococcus* spp. suggests the possibility of minor infections that do not induce inflammation in Treatment C and Treatment D. No significant differences were observed between Treatment C and Treatment D in BC2 or bacterial isolation rates. Additionally, the isolation rate of other environmental bacteria did not differ between the two groups, suggesting that the increase in BC2 was primarily attributable to *Staphylococcus* spp. Since various bacteria, including *Staphylococcus* spp., establish a bacterial flora within the teat canal and teat end [23], the bacteria isolated from the milk could have originated from both the cow and the barn environment. This finding underscores the importance of post-dipping in reducing the risk of intramammary infection. Statistical analysis showed that the teat score may influence viable bacterial counts (BC2 in Experiment 1) and SCC (Experiment 2). However, a relationship could not be clearly established due to variability in teat scores among the cows in each experiment and the limited number of cases. Although this study did not clarify how teat scores relate to bacterial counts in foremilk, the results suggest that the omission of teat disinfection may increase the risk of intramammary infection. Therefore, ETS cannot be considered a substitute for teat disinfection.

## 5. Conclusions

ETS application did not provide any advantage over post-dipping after milking. Furthermore, there was an increased risk of intramammary infection when ETS application was substituted for teat orifice disinfection treatments (pre-dip and post-dip). Further research is needed to draw definitive conclusions about the adhesion, barrier performance, and preventive effect of ETS against intramammary infection under field conditions, as these may be influenced by dose and climate. Detailed microbial dynamics of intramammary infection may become more evident using high-throughput sequencing technologies.

## Figures and Tables

**Table 1:** Characteristics of cows used in each experiment, showing interventions and ETS dropout rates in different treatments.

		Cow/Quarters Status					Intervention			
Experiment	Treatment	Parity (Mean ± S.D.)	log SCC (Cells/mL) (Mean ± S.D.)	Teat Score *			Pre-Dip	Post-Dip	ETS Application	ETS Dropout (%)
				1	2	3				
1	A (cont.)	1.1 ± 0.4	4.76 ± 4.59	0	11	8	Yes	Yes	No	0 (0/76)
	B						Yes	No	Yes	14.5 (11/76)
2	C	1.0 ± 0.0	4.70 ± 4.46	7	4	11	Yes	No	Yes	30.7 (27/88)
	D						No	No	Yes	23.9 (21/88)

*: Teat score was determined based on Mein et al. [17]. ETS: external teat sealant; SCC: somatic cell counts; Treatment A: pre-dip and post-dip treatments; Treatment B: ETS application instead of post-dip; Treatment C: same as group B; Treatment D: ETS treatment only.

**Table 2:** Variables measured after intervention (mean ± standard deviation).

Experiment	Treatment	n	Variables			
			logATP (RLU)	logSCC (cells/mL)	logBC1 (CFU/mL)	logBC2 (CFU/mL)
1	A (cont.)	76	3.57 ± 0.21	4.60 ± 0.64	3.47 ± 0.59	3.33 ± 0.49
	B	65	3.14 ± 0.49	4.49 ± 0.71	3.23 ± 0.86	3.15 ± 1.00
2	C	61	3.41 ± 0.38	4.14 ± 0.42	3.03 ± 1.16	3.34 ± 0.96
	D	67	3.67 ± 0.23	4.21 ± 0.55	3.57 ± 0.47	3.73 ± 0.32

RLU: Relative light unit; ATP: Adenosine triphosphate; SCC: Somatic cell counts; BC1: Bacterial counts in first foremilk; BC2: Bacterial counts in milk just before the milking unit was attached.

**Table 3:** Statistical analysis of each variable using a mixed-effects model.

**Experiment 1**				
	***p*** **-value**			
**Fixed effects**	**ATP**	**SCC**	BC1 *	**BC2**
Teat score	0.38	0.23	0.17	0.02
Parity	0.32	0.85	0.11	0.35
Sequence	0.69	0.17	NT	0.86
Treatment	<0.01	0.34 **	0.02	0.17 **
Period	0.22	0.04	NT	0.04
**Experiment 2**				
	***p*** **-value**			
**Fixed effects**	**ATP**	SCC *	BC1 *	**BC2**
Teat score	0.84	<0.01	0.16	0.13
Sequence	0.79	NT	0.21	0.23
Treatment	0.25 **	0.11	0.49 **	0.78 **
Period	<0.01	NT	0.03	0.07

*: Paired *t*-tests based on Period 1 data to avoid carryover effect (except for teat score in Experiment 2, which was analyzed by ANOVA). Sequence and period were not tested. **: Paired *t*-tests based on Period 1 data to avoid period effect. ATP: adenosine triphosphate; SCC: somatic cell counts; BC1: bacterial counts in first foremilk; BC2: bacterial counts in milk just before the milking unit was attached; NT: Not tested.

## Data Availability

The data presented in this study are available on request from the corresponding author. Because of their containing information that could compromise the privacy of research participants.

## Abbreviations

The following abbreviations are used in this manuscript:

ATP	adenosine triphosphate
BC1	bacterial counts in first foremilk
BC2	bacterial counts in milk just before the milking unit was attached
ETS	external teat sealant
RLU	relative light unit
SCC	somatic cell counts
